# A Distal to Proximal Gradient of Human Choroid Plexus Development, with Antagonistic Expression of Glut1 and AQP1 in Mature Cells vs. Calbindin and PCNA in Proliferative Cells

**DOI:** 10.3389/fnana.2016.00087

**Published:** 2016-09-23

**Authors:** Leandro Castañeyra-Ruiz, Ibrahim González-Marrero, Luis G. Hernández-Abad, Emilia M. Carmona-Calero, Gundela Meyer, Agustín Castañeyra-Perdomo

**Affiliations:** ^1^Department of Neurosurgery, School of Medicine, Washington University in Saint LouisSt. Louis, MO, USA; ^2^Unidad de Anatomía, Departamento de Ciencias Médicas Básicas, Facultad de Medicina, Universidad de La LagunaLa Laguna, Spain; ^3^Instituto de Investigación y Ciencia de Puerto de Rosario (INIPRO)Fuerteventura, Spain

**Keywords:** aquaporin-1, Glut1, calbindin, PCNA, choroid plexus development

## Abstract

The choroid plexuses (ChP) are highly vascularized tissues suspended from each of the cerebral ventricles. Their main function is to secret cerebrospinal fluid (CSF) that fills the ventricles and the subarachnoid spaces, forming a crucial system for the development and maintenance of the CNS. However, despite the essential role of the ChP–CSF system to regulate the CNS in a global manner, it still remains one of the most understudied areas in neurobiology. Here we define by immunohistochemistry the expression of different proteins involved in the maturation and functionality of the ChP from the late embryological period to maturity. We found an opposite gradient of expression between aquaporin 1 (AQP1) and glucose transporter 1 (Glut 1) that define functional maturation in the ChP periphery, and proliferating cell nuclear antigen (PCNA) and calbindin (CB), present in the ChP root zone with proliferative activity. We conclude that the maturation of the ChP matures from distal to proximal, starting in the areas nearest to the cortex, expressing in the distal, mature areas AQP1 and Glut1 (related to ChP functionality to support cortex development), and in the proximal immature areas (ChP root) CB and PCNA related to progenitor activity and proliferation.

## Introduction

The choroid plexuses (ChP) are highly vascularized tissues suspended from each of the cerebral ventricles. They comprise a central stroma with numerous blood vessels covered by a single layer of specialized epithelial cells (the choroid epithelium) resting on a thick basement membrane (Johanson et al., 2008; González-Marrero et al., 2012, 2015). These specialized organs have two major functions: to act as a diffusion barrier between the blood and the cerebrospinal fluid (CSF; the site of the blood–CSF barrier) allowing or inhibiting the passing of proteins as a growth factors, and to secrete CSF that fills the ventricles and the subarachnoid spaces. The ChP is considered a crucial system for the development and maintenance of the CNS (Davson and Segal, 1995; Johanson, 1995; Lun et al., 2015).

However, during brain development these functions might be quite different from those in the adult brain, given that both, the size of the ChP and the size of the CSF-filled brain ventricles in relation to brain size, are much larger in development than in the adult, and that in the early stages of brain development no vessels are present within the brain parenchyma, the ChP may be of crucial importance for supplying developmentally essential materials from the blood to the brain at that time point (Klosovskii, 1963; Ek et al., 2003; Johansson et al., 2005).

Prior to ChP formation, a primitive ventricular system is first formed when the neural tube closes and amniotic fluid is trapped within the central canal. This closure is associated with a subsequent rise of intraventricular fluid pressure and increase in CSF protein concentration, and coincides with a start of rapid brain enlargement. Desmond (1985) and Desmond and Jacobson (1977) suggested that the ventricular fluid might provide an essential pressure force for normal brain expansion and morphogenesis, with the ChP taking over this function.

The choroid plexus epithelium (ChPE) forms as an invagination of the neuroepithelium which differentiates into more specialized epithelial cells following four stages. Initially in stage I, these cells form a pseudostratified epithelium, in stage II, they form a simple high columnar epithelium, in stage III the epithelial cells flatten and widen forming a squamous epithelium presenting apical or centrally positioned nuclei and increased levels of glycogen content. In most species this stage can be followed by stage IV, which is characterized by lower glycogen content and basally positioned nuclei (Dziegielewska et al., 2001; Johansson et al., 2005; Lun et al., 2015).

However, despite the essential role of the ChP–CSF system to regulate the CNS in a global manner, it still remains one of the most understudied areas in neurobiology (Lun et al., 2015).

In order to understand the development of the human ChP–CSF system, we examined the expression of different key proteins involved in the functionality and maturation of ChP during the fetal cortex development.

1. Aquaporin 1 (AQP1) is a water channel protein that makes a substantial contribution to CSF production and is expressed in early stages of brain development (Johansson et al., 2005; Gömöri et al., 2006). This expression follows a temporo-spatial progression during fetal life, starting in ChPE of the fourth ventricle, then in the lateral and finally in the third ventricle (3V), following the same order as the regional histology of ChPE in developing fetal ventricles (Johansson et al., 2005), being a reliable marker of the functionality of the ChP.
2. Glucose transporter 1 (Glut 1), is a uniporter protein that transports glucose from the extracellular medium into cells (Bolz et al., 1996; Kapoor et al., 2016). Given the importance of the presence of glycogen for ChPE cell maturation, we propose Glut1 as good marker for understanding the ChP development.
3. Calcium-binding protein D-28K (Calbindin, CB), is a member of the large family of intracellular calcium-binding proteins related to calmodulin and troponin-C. CB plays biological roles in calcium regulation (transport, uptake, calcification of bone and teeth) and calcium related signaling in neurons, and transiently in embryological development. CB stains a regionalized area in human brain development, defining the neuroepithelium of the cortical hem, the medial boundary of the cortex with the ChP, at 6 gestational weeks (GW), and is therefore a reliable marker for the human ChP in the early development. (Meyer, 2010; González-Gómez and Meyer, 2014).
4. Proliferating cell nuclear antigen (PCNA) is a cofactor of DNA polymerases that encircles DNA and orchestrates several of the replication functions by recruiting crucial mitotic players (Moldovan et al., 2007). It is only expressed in proliferating cells, and thus allows us to understand the mitotic pattern of the developing ChPE.

Taking into account the ultrastructural studies that have been performed on the ChPs from all four ventricles, where they all undergo identical developmental stages (el-Gammal, 1981, 1983; Ek et al., 2003) and that, this work is focused in the cortical brain development, the aim of this work is to define immunohistochemically the expression of different proteins involved in the maturation and possible functionality of the telencephalic ChPs from the late embryological period to the end of the ChP development.

## Materials and Methods

Fourteen late embryonic and fetal brains at 7, 8, 9, 10(3), 13, 15(2), 16(2), 20, 21 and 25 GW, one newborn infant and three adult men brains (59, 63 and 73 years old respectively) were used in this work. The brains were from the collection of the Department of Anatomy at the University of La Laguna. Parents had given written informed consent. The ethical committee of the University of La Laguna and the University Hospital of the Canary Islands approved and supervised the study.

The brains were processed using the following standardized protocol: fixation in formaldehyde, post fixation in Bouin or paraformaldehyde for 24 h, dehydration and paraffin embedding. The brains were then cut in four (A, B, C and D) coronal series of 10 microns thick sections. The A series were stained with hematoxylin-eosin (H-E) and the B, C and D series were used for immunohistochemistry.

### Immunocytochemistry and Immunofluorescence

Tissue sections were deparaffinized in xylene, hydrated through descending ethanol and washed in Tris-buffer saline (TBS 0.05 M; pH 7.6). For immunocytochemistry, sections were incubated in the primary antibodies; rabbit anti-AQP1 (Sigma-Aldrich) at 1:1000, rabbit anti-CB 38a (Swant) at 1:7000, mouse anti-PCNA (Abcam) at 1:200 and mouse anti-Glut1(Abcam) at 1:250 overnight in a humid chamber and Goat anti-rabbit and mouse IgG conjugate antibodies (Invitrogen) were used as secondary antibodies. In light microscopy, the “DAKO” Strept ABC complex/HRP Duet, Mouse/Rabbit procedure was used and the peroxidase reaction product visualized with diaminobenzidine intensified with nickel. For immunofluorescence, sections were incubated with the following secondary antibodies; Cyanine 3 (Cy3; red) goat anti-rabbit IgG (Invitrogen) and alexa-fluor 488 (green; Invitrogen). After washes, samples were mounted in Vectashield Medium (Vector Laboratories Inc.) for viewing with a confocal microscope (FV 1000 Olympus).

## Results

**Figure 1:**
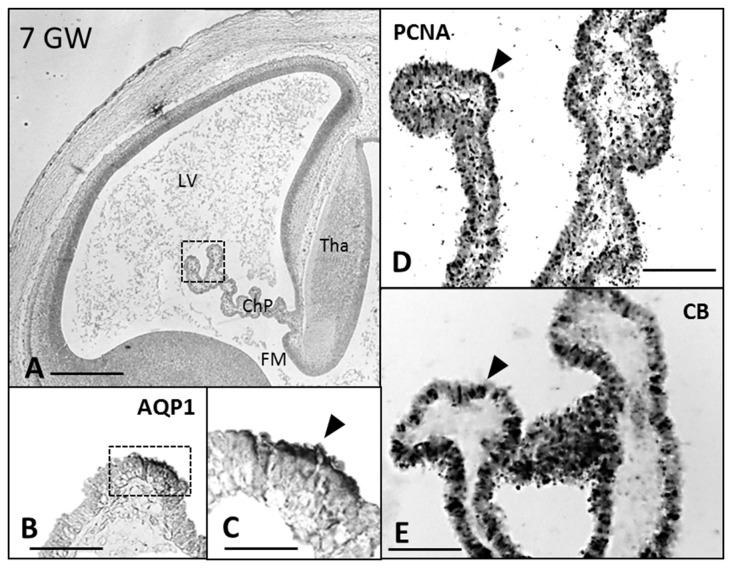
**Paraffin coronal section of human brain embryo-fetus at seven gestational weeks (GW) showing LV and ChP marked with anti-AQP1, anti-PCNA and anti-CB. (A)** Panoramic view showing the AQP1 immunoreaction, bar = 600 μm; **(B,C)** detailed magnification of the distal ChP in **(A)**, where the expression of AQP1 is localized in the apical cell pole of the ChP epithelium. **(D)** PCNA immunoreaction. **(E)** anti-CB expression, bar = 40 μm in **(C)** and 80 μm in (**B,D,E**; Arrowheads indicate the expression of proteins). AQP1****, aquaporin 1; CB, calbindin; PCNA, proliferating cell nuclear antigen; LV, lateral ventricle; FM, foramen of monro; Tha, thalamus; ChP, choroid plexus.

**Figure 2:**
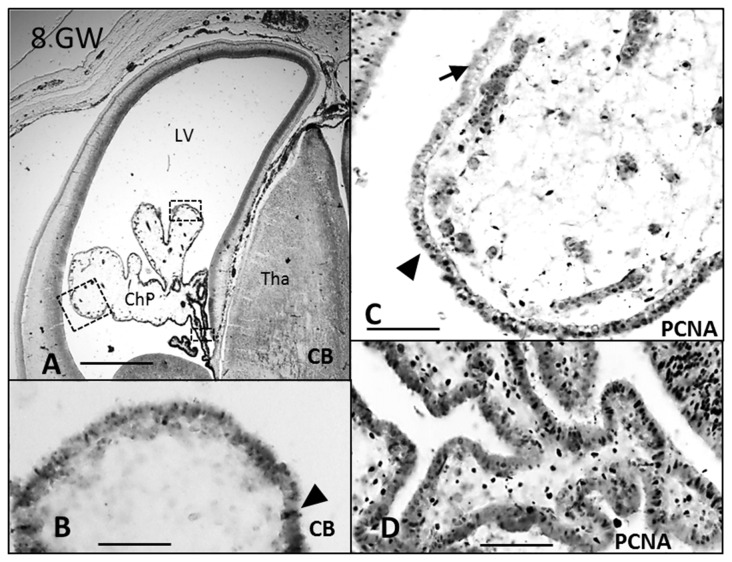
**Paraffin coronal section of human brain fetus at 8 GW showing LV and ChP marked with anti-CB and anti-PCNA. (A)** Panoramic view showing the anti-CB immunoreaction in epithelial cells of the ChP, bar = 600 μm; **(B)** detailed magnification of area frame in **(A)**, bar = 80 μm; **(C,D)** anti-PCNA expression in the distal and proximal areas of the ChP, bar = 80 μm in **(C)** and 60 μm in (**D**; Arrowheads indicate the expression of CB and PCNA, and arrows indicate where its expression is lost). CB, calbindin; PCNA, proliferating cell nuclear antigen; LV, lateral ventricle; Tha, thalamus; ChP, choroid plexus.

**Figure 3:**
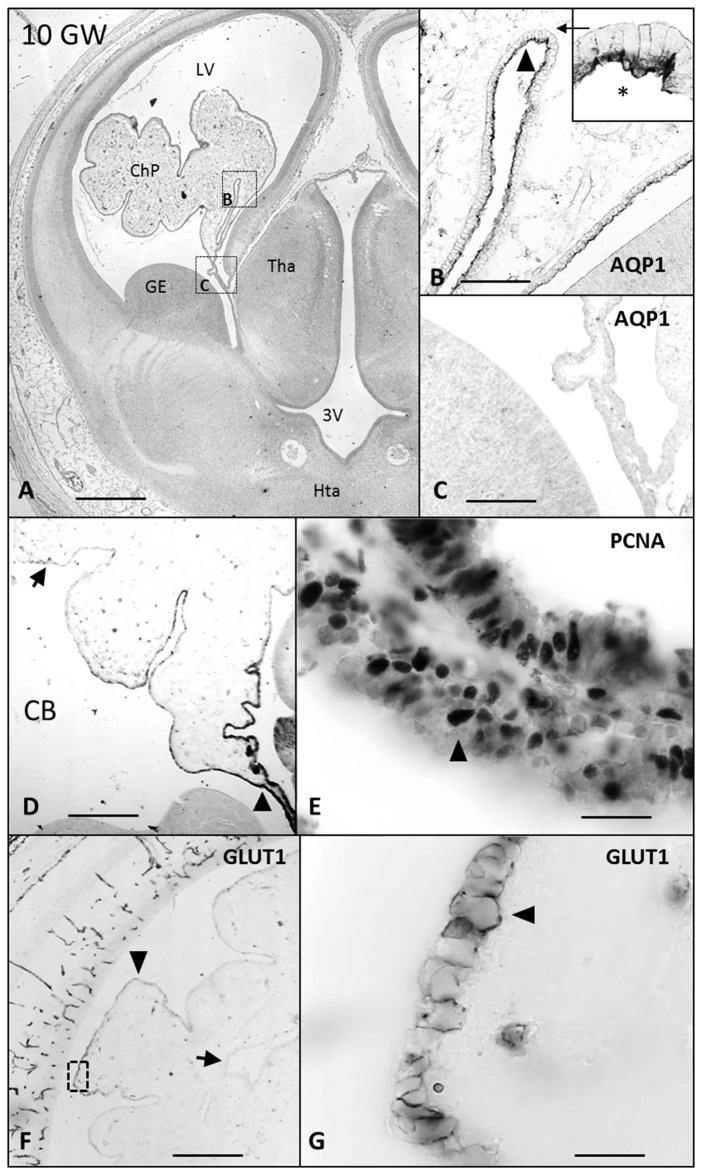
**Paraffin coronal section of human brain fetus at 10 GW showing LV and ChP, marked with anti-AQP1, anti-PCNA and Glut1. (A)** Panoramic view showing the anti-AQP1 bar = 800 μm; **(B)** detailed magnification of area frame in **(A)**, asterisk indicates the expression of AQP1 in the apical pole of ChPE, bar = 80 μm; **(C)** undetectable AQP1 immunoreaction of the epithelial cells of the ChP root, bar = 80 μm; **(D)** panoramic view showing the anti-CB immunoreaction that is lost in the distal choroid plexus epithelium (ChPE) and remains in the stalk, bar = 200 μm; **(E)** detailed of ChP root marked with anti-PCNA, bar = 30 μm. **(F)** Panoramic view showing the anti-Glut1 only expressed in the distal areas of the ChP, bar = 200 μm and **(G)** detailed magnification of area frame in **(F)** showing the expression of Glut1 located in the basolateral membrane of the ChPE, bar = 30 μm (Arrowheads indicate the expression of CB, PCNA, Glut1 and AQP1, and arrows indicate where its expression is lost or have had no place). AQP1, aquaporin 1; CB, calbindin; PCNA, proliferating cell nuclear antigen; Glut1, Glucose transporter 1; LV, lateral ventricle; FM, foramen of monro; Tha, thalamus; 3V, third ventricle; GE, ganglionic eminence; Hta, hypothalamus.

CB and PCNA were detected in the cytosol and in the nucleus, respectively, at 7 GW (Figure 1) in the entire ChP anlage, but at 8 and 10 GW this expression was lost from distal to proximal (Figures 2B,C, 3D), remaining only in the mitotically active zones of the ChP stalk (Figures 2D, 3E). At 20 GW this expression was no longer observed.

AQP1 expression was first observed in the ChP at 7 GW (Figures 1B,C). This expression begins in the distal areas, away from the choroidal stem, forming small immunoreactive foci. At 7 and 8 GW, the ChP was mainly a pseudostratified epithelium and AQP1 was expressed only in the apical pole of the ChPE cells in contact with the ventricle. At 10 GW, the size of the ChP was greatly increased, filling most of the ventricular cavities. Moreover, the presence of connective tissue and a few choroidal folds were also observed. AQP1 expression was detected at the apical pole of the ChPE cells (Figures 3A,B) but there was no immunoreactivity in the choroidal stem and adjacent areas, which were, in turn, positive for the mitotic markers (PCNA; Figures 3C,E).

**Figure 4:**
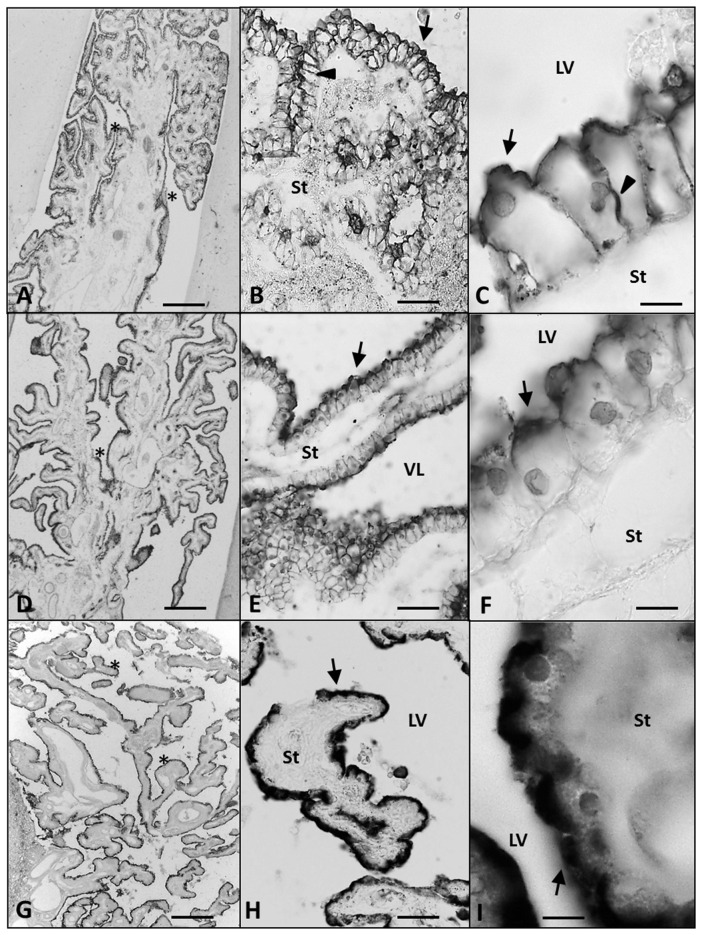
**Paraffin coronal section of human brain fetus at 21 (A–C) and 25 (D–F) GW, and a 63 year old human brain (G–I) showing LV and ChPE marked with anti-AQP1 (arrow). (A)** Panoramic view of ChP showing anti-AQP1, bar = 200 μm at 21 GW; **(B)** magnification of area frame in **(A)**, bar = 60 μm; **(C)** detailed magnification at 21 GW showing detectable AQP1 immunoreaction in the apical, lateral and basal domain of the ChP epithelial, bar = 10 μm; **(D)** panoramic view showing the anti-AQP1 in ChP at 25 GW, bar = 200 μm; **(E,F)** ChP magnification showing anti-AQP1 mainly in the apical pole of the epithelial cells, bar = 60 and 10 μm respectively; **(G–I)** show anti-AQP1 mainly in the apical pole of the ChP epithelial cells of the 63 year old man, bar = 200, 60 and 10 μm respectively: (arrows indicate the expression of AQP1 in the apical pole of the ChP epithelium, arrowheads indicate its expression in the basolateral domain of the epithelial cells and the asterisks indicate the folds of the ChP). AQP1, aquaporin 1; LV, lateral ventricle; St, stroma.

At 21 GW (Figures 4A–C), AQP1 expression was not only detected in the apical membrane of the ChPE cells, but also in their basolateral membrane even reaching the basal pole. However, at 25 GW, when choroidal folds had increased in numbers, AQP1 expression was concentrated again limited at the apical pole (Figures 4D–F). In the adult, we found that the expression of AQP1 was not only in the apical pole but also in the supranuclear domain. (Figures 4G–I).

Glut1 was detected for first time at 9 GW in the basolateral membrane of the distal area of ChP (Figures 3F,G), presenting no changes during the entire gestational progress in its intracellular location.

**Figure 5:**
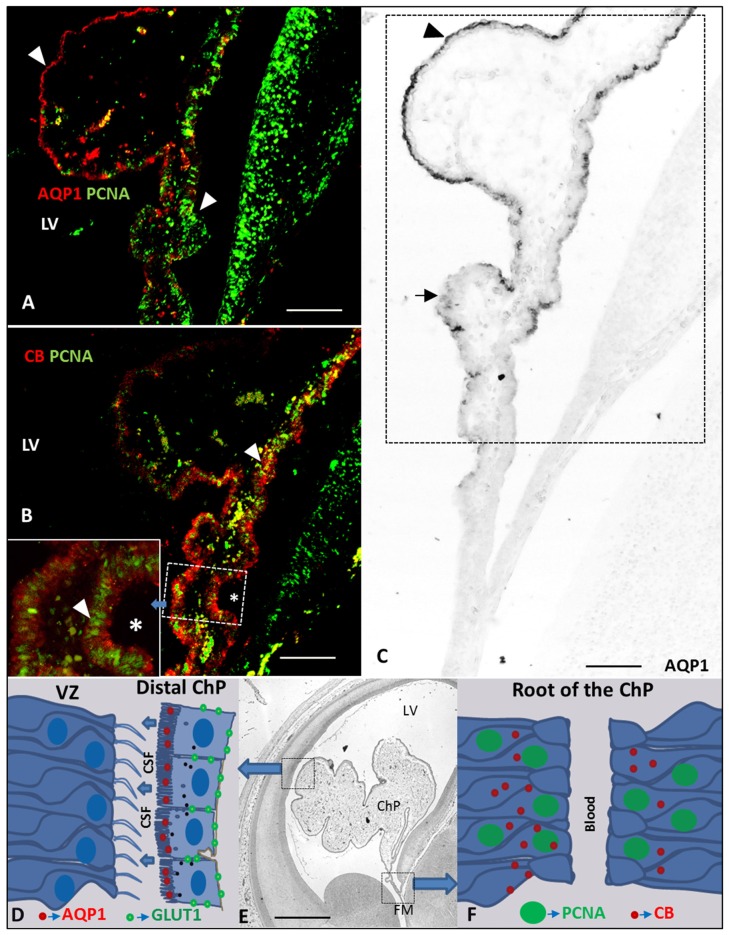
**Drawing and Paraffin coronal section of the brain of a human fetus at 10 GW. (A)** ChP (frame of C) marked with anti-AQP1 and anti-PCNA, bar 100 = μm; **(B)** ChP (frame of C) marked with anti-CB and anti-PCNA, asterisk indicate a frame magnification of the root of the ChP, bar 100 μm; **(C)** panoramic view showing of the ChP marked with anti-AQP1, bar = 100 μm; **(D)** drawing of AQP1 and GLUT1 expression at latero-distal part of the ChP and neuroepithelium of the ventricular zone (VZ) of the LV; **(E)** panoramic coronal view showing the whole LV and ChP, bar = 800 μm; **(F)** drawing of PCNA and CB expression at ChP root (proximal or medial part of ChP; Arrowheads indicate the expression of PCNA, AQP1 and CB and arrows indicate where its expression is not present). AQP1, aquaporin 1; CB, calbindin; PCNA, proliferating cell nuclear antigen; GLUT1, Glucose transporter 1; LV, lateral ventricle; FM, foramen of monro; Tha, thalamus; 3V, third ventricle; GE ganglionic eminence; Hta, hypothalamus.

We thus found that the expression of AQP1 and CB follow an opposite gradient, with AQP1 following a distal-proximal expression in ChPE cells that were negative for PCNA (Figures 5A,B); CB was expressed throughout the ChP anlage at 7 GW, and was thereafter restricted to the proximal ChP root, mainly in proliferating cells (Figures 2A, 4A,C,D).

## Discussion

At present, only two reports have studied AQP1 expression in human brain development. Johansson et al. (2005) examined fetuses from 5.5 to 23 GW, whereas Gömöri et al. (2006) studied fetuses from 14 to 40 GW. We detect first expression of AQP1 in ChP at 7 GW, being located only in the apical pole of ChPE in the beginning of the stage II (Table 1). Our findings basically concur with Johansson et al. (2005), who describe that towards the end of the embryonic period (CRL: 28 mm) at 8 GW, the majority of the ChP cells show positive AQP1 staining of the apical cell membrane. However, we detected AQP1 even earlier, at 7GW, in small foci of positive ChE cells, at the distal end of the ChP, but not in the mitotically active choroid stem. A few weeks later, at 10 GW, we found immunostaining in the entire ChP except in the stem. We thus detect a temporo-spatial pattern in AQP1 expression that starts in small foci distally and progresses proximally toward the ChP stem. This pattern parallels histological differentiation of the ChP which follows the same distal-to -proximal gradient (el-Gammal, 1981, 1983; Zheng and Chodobski, 2005; Lun et al., 2015). The cells at the stem or root of the ChP are less mature and remain mitotically active for a longer period compared to the more distal ChP folds. Growth of the ChP is thus initiated at the root, where most mitotic figures are found, and where the cells show a pseudostratified appearance until later in development (Knudsen, 1964; Tennyson and Pappas, 1964; Ek et al., 2003; Zheng and Chodobski, 2005).

**Table 1:** Correlative table of the expression (*) or not expression (−) of the proliferating cell nuclear antigen (PCNA), calbindin (CB), aquaporin 1 (AQP1) and glucose transporter 1 (Glut 1) in the different stages of the choroid plexuses (ChP) development.

Marker	Stage I	Stage II	Stage III	Stage IV
PCNA	*	−	−	−
CB	*	*	−	−
AQP1	−	*	*	*
Glut 1	−	−	*	*

Glut 1 follows the same pattern of expression than AQP1 but delayed in time. We first observed the Glut 1 expression at 9 GW in the distal areas of the ChP progressing to the proximal areas, indicating different stages of the maturation and functionality of the ChP and corresponding with the stage III of the development of the ChP (Table 1). Exactly the opposite happened with CB and PCNA: where these proteins were expressed, there was no expression of AQP1 and Glut1or was scarcely expressed (Figures 5C–F), suggesting they are markers for ChP progenitor cells. Since AQP1 and Glut1 are involved in the maturation and production of the CSF, our findings suggest that the ChP starts to be active from very early stages of brain development in the distal areas of the ChP, close and almost adjacent to the cortex, as a possible source of growth factors for cortex development because of the its low vascularization in early fetal development (Klosovskii, 1963; Ek et al., 2003; Johansson et al., 2005). At the same time the proximal root of the ChP still keeps its progenitor activity which allows the ChP to keep growing in order to be able to adapt the ChP size to the growth of the lateral ventricle (LVs) in telencephalic development.

From the late embryonic age to adulthood we found dynamic changes in AQP1 expression. Initially it appeared only in the apical cell membranes, at midgestation also in the basolateral membranes and, after 25 GW, expression was exclusively in the apical membrane, as also described by Johansson et al. (2005) and Gömöri et al. (2006). In the adulthood, we also found a supranuclear expression of the AQP1, recently described for our group (Castañeyra-Ruiz et al., 2016). Therefore the polarization of AQP1 expression undergoes dynamic changes along gestation, possibly related with CSF production that might provide different ventricular pressure forces to support brain expansion at distinct moments of corticogenesis.

AQP1 expression appears for the first time in the ChP at 7 GW following a distal to proximal pattern of expression, and then changes dynamically during development, adjusting ventricular size and CSF production according to the requirements of the expanding brain. Glut 1 appears for the first time at 9 GW, following the same gradient of expression than AQP1 although delayed.

CB and PCNA follow a decreasing pattern of expression from distal to proximal indicating the ongoing progenitor activity of the ChP stalk during the first half of gestation and mainly related to the stage I of the ChP development (Table 1). In conclusion, the telencephalic ChP differentiates from distal to proximal, starting to mature in the areas nearest to the cortex, and expressing proteins such as AQP1 and Glut1, while still expressing CB and PCNA in the root, indicating immaturity and proliferation.

## Author Contributions

LC-R: designed the study; collected, analyzed and interpreted data; revised the manuscript. IG-M: designed the study; collected, analyzed and interpreted data. LGH-A, EMC-C and GM collected and analyzed data; revised the manuscript. AC-P: designed the study; revised the manuscript. All authors approved the final version and agreed to be accountable for all aspects of the work.

## Funding

This study was supported by the research projects from: “Fundación Canaria Instituto de Investigacion y Ciencias de Puerto del Rosario” (INIPRO) project no. 01/10 and “Universidad de la Laguna” (ULL) Project no. 2013/0001341, and “La Fundación Canaria Doctor Manuel Morales”.

## Conflict of Interest Statement

The authors declare that the research was conducted in the absence of any commercial or financial relationships that could be construed as a potential conflict of interest.
